# A diagnostic dilemma following risk-reducing surgery for *BRCA1 *mutation – a case report of primary papillary serous carcinoma presenting as sigmoid cancer

**DOI:** 10.1186/1477-7819-5-102

**Published:** 2007-09-12

**Authors:** Manish Chand, Patrick J Moore, Andrew D Clarke, Guy F Nash, Tamas Hickisk

**Affiliations:** 1 Poole General Hospital, Longfleet Road, Poole, Dorset, BH152JB, UK

## Abstract

**Background:**

Women that carry germ-line mutations for *BRCA1 *or *BRCA2 *genes are at an increased risk of developing breast, ovarian and peritoneal cancer. Primary peritoneal carcinoma is a rare tumour histologically identical to papillary serous ovarian carcinoma. Risk-reducing surgery in the form of mastectomy and oophorectomy in premenopausal women has been recommended to prevent breast and ovarian cancer occurrence and decrease the risk of developing primary peritoneal cancer.

**Case presentation:**

We present a case report of a woman with a strong family history of breast cancer who underwent risk-reducing surgery in the form of bilateral salpingo-oophorectomy following a mastectomy for a right-sided breast tumour. Following the finding of a *BRCA1 *mutation, a prophylactic left-sided mastectomy was performed. After remaining well for twenty-seven years, she presented with rectal bleeding and altered bowel habit, and was found to have a secondary cancer of the sigmoid colon. She was finally diagnosed with primary papillary serous carcinoma of the peritoneum (PSCP).

**Conclusion:**

PSCP can present many years after risk-reducing surgery and be difficult to detect. Surveillance remains the best course of management for patients with known *BRCA *mutations.

## Background

Women with *BRCA1 *or *BRCA2 *germ-line mutations are at an increased risk of developing tumours of the breast, ovary and peritoneum. The cumulative lifetime risk of invasive breast cancer for women with either mutation is between 60–85% whereas this is between 15–65% for epithelial ovarian cancer [1-3]. Management strategies for genetically susceptible women include genetic counselling, chemoprevention, radiological and tumour-marker surveillance, and risk-reducing surgery such as mastectomy and bilateral salpingo-oophorectomy.

Primary papillary serous carcinoma of the peritoneum (PSCP) is a rare tumour found predominantly in elderly and post-menopausal women [4]. It shares many features with its ovarian analogue, serous ovarian papillary carcinoma, making diagnosis less than straightforward by both radiological imaging and histological analysis. Although the pathogenesis of PSCP remains unclear, familial studies have led to the inclusion of peritoneal carcinoma in the hereditary breast ovarian cancer (HBOC) syndrome, which also includes Fallopian tube neoplasms [5,6].

The authors present an interesting case of primary peritoneal cancer. This was diagnosed twenty-seven years after risk-reducing surgery (ovarian ablation and mastectomy) for a *BRCA1 *mutation in the context of breast disease management. Following investigation for bowel related symptoms, she was found to have a secondary sigmoid cancer of indeterminate origin. After undergoing a laparotomy and resection of this lesion, a diagnosis of PSCP was reached.

## Case presentation

A 33-year old Caucasian woman was diagnosed with adenocarcinoma of the right breast in 1976 following the discovery of a breast lump. She underwent a right-sided mastectomy without adjuvant therapy. Two years later at age 35, back pain prompted a bone scan which revealed a 'hot spot' in the region of the lumbar spine. This was thought to represent spinal metastases and the patient received local radiotherapy. After counselling for risk-reducing surgery, the patient chose to undergo a bilateral salpingo-oophorectomy, but declined a proposed hysterectomy.

There was a strong family history of breast disease with both her mother and maternal grandmother diagnosed with breast cancer at 34 years and 41 years, respectively. There were no other known cancers in her family. In 2004, aged 62 years, the patient underwent genetic testing, which uncovered a *BRCA1 *mutation (185delAG). A prophylactic left-sided mastectomy was subsequently performed.

**Figure 1:**
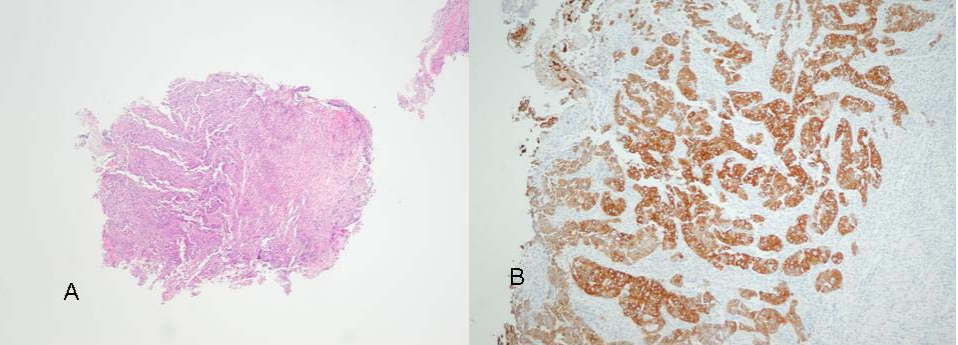
1a)Sigmoid colon biopsy, and 1b) with CK7 stain demonstrating features of ovarian-type adenocarcinoma.

Aged 64 years, the patient presented complaining of change in bowel habit, faecal leakage, urgency and left-sided abdominal discomfort. Examination revealed a tender, palpable fullness in the left iliac fossa. Initial routine blood tests showed haemoglobin of 11.7 g/dl with a normal mean cell volume and no abnormalities of electrolytes, liver or kidney function. Tumour markers revealed carcinoembryonic antigen (CEA) 3 ug/L and CA125 3 Ku/L (both within normal limits). At colonoscopy, a necrotic lesion was seen in the proximal sigmoid colon. Biopsies confirmed a very poorly differentiated papillary adenocarcinoma of the sigmoid, the immunoprofile of which favoured ovarian origin rather than colonic; being highly positive to CK7, while negative to CK20 (Figures 1a and 1b). In addition, there was some faint staining for ER but PR staining was negative. A staging CT scan excluded macroscopic metastases and reported a large bowel-related mass measuring 68 mm by 67 mm, contiguous with the left lateral aspect of the bladder with no associated pathological lymphadenopathy. The radiological appearance suggested a malignancy of colonic origin.

**Figure 2:**
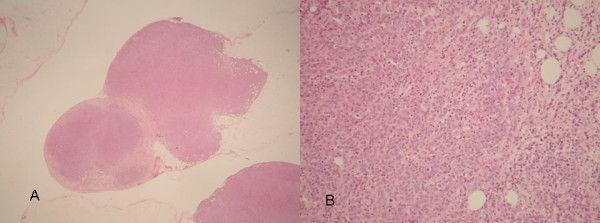
A) and B) Microphotograph of peritoneal nodules.

**Figure 3:**
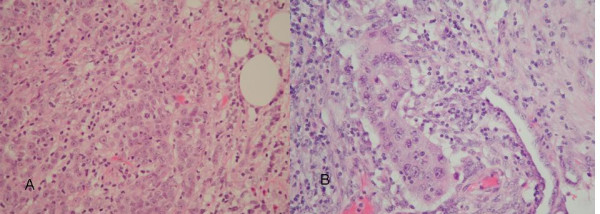
A) and B) Histological section of sigmoid lesion taken at laparotomy.

Laparotomy findings included a bulky tumour adherent to the left pelvic brim and pelvic side-wall, as well as two separate peritoneal deposits, one within the pelvis and one at the terminal ileum. There was no further pathology noted. The patient underwent an en bloc resection of sigmoid colon, bladder, omentum, left pelvic side wall and uterus. The histology of the surgical specimens revealed peritoneal nodules of poorly differentiated papillary adenocarcinoma (Figures 2a and 2b), a normal atrophic uterus with no evidence of tumour nor Fallopian tube remnants, and a poorly differentiated papillary adenocarcinoma of the large bowel which penetrated the bowel wall and had invaded adherent bladder (Figures 3a and 3b). There was associated extramural venous invasion but no metastatic lymph nodes (total of 3 found). When the specimens were reviewed in a multidisciplinary setting, incorporating colorectal surgeons and gynaecologists, it was concluded that they represented a primary peritoneal carcinoma of high grade. A post-operative CA125 titre performed within 2 weeks of the operation was 3 Ku/L and subsequent titres over the following 4 months were between 3 and 4 Ku/L. She is currently receiving chemotherapy treatment.

## Discussion

Mutations in *BRCA1 *gene (and *BRCA2*) are associated with inherited breast and ovarian cancer, although the exact nature of this tissue specificity is incompletely understood. The prevalence of mutations of *BRCA1 *gene in the general population is between 0.07% and 0.24% and between 0.14% and 0.22% for *BRCA2 *although this is higher in Ashkenazi Jews [7,8]. The risk of primary peritoneal carcinoma is also increased in patients carrying *BRCA1 *(and *BRCA2*) mutations with a cumulative lifetime risk of between 1.3–20% [6,9,10]. This is in contrast with invasion of the peritoneum by carcinomatous metastasis is far more common.

The development of PSCP is thought to originate de novo from the peritoneal lining of the pelvis and abdomen. However, it is histologically indistinguishable from its ovarian analogue and may develop in women years after a prophylactic oophorectomy [11]. Two theories have been proposed to explain these similarities. The first suggests that peritoneal carcinoma originates from nests of ovarian tissue remnants that are left within the peritoneum during embryonic descent of the female gonads. The second suggests that both ovarian and peritoneal tissue (with a common mesodermal origin), have mullerian potential [12,13].

Diagnosing PSCP can be particularly challenging as most peritoneal carcinomas present late and are difficult to detect both clinically and radiologically. There are certain CT features which are specific to peritoneal disease, but these are subtle and may only help in differentiating from other similar lesions such as mesothelioma and metastatic disease [14]. There is some role for serial tumour marker measurements (CA-125) but this may be misleading. Although it is not uncommon to have a low initial CA-125 titre, this value would be expected to rise following a debulking procedure. However, this did not occur in our case.

In the case report the patient had undergone ovarian ablation as a form of risk-reduction surgery during the management of breast cancer. The genetic associations were not known at the time but the surgery acted indirectly as prophylaxis for future ovarian and peritoneal cancer. The initial dilemmas with this patient were in understanding the origin of the sigmoid tumour. The clinical picture, colonoscopy findings and CT images suggested a primary colonic pathology which was in contrast to the histology results favouring an ovarian origin. This was made all the more confusing as the patient had previously undergone a bilateral salpingo-oophorectomy and serum CA125 titres were normal. The fact that the sigmoid colon had become a site of tumour spread was not surprising due to the intimate anatomical relationship of peritoneum and sigmoid colon.

This case highlights the difficulties faced by patients with *BRCA *mutations as they are at an increased risk of cancer from several sources. In addition to the risks of those cancers which make up the HBOC syndrome, there have been lower but significant risks reported for other cancers, including pancreas and stomach [15,16]. When considering surgical prophylaxis, bilateral salpingo-oophorectomy and total hysterectomy is the most effective procedure to reduce the risk of development of ovarian and peritoneal carcinoma [17]. The estimated risk reduction is between 80–85% for ovarian, Fallopian tube and peritoneal cancer [10,18]. It is important to appreciate however, that in the case described, the patient chose not to have a hysterectomy and that the surgery was performed in the context of a breast tumour and not ovarian and peritoneal disease. There may have been retained Fallopian tube remnants which could have been a potential source of malignancy. For risk-reduction surgery to be most beneficial in the context of ovarian, Fallopian tube and peritoneal cancer, surgery should include bilateral salpingo-oophorectomy and hysterectomy with the surgical specimens to be examined in fine detail to exclude the presence of microscopic disease. The estimated risk for developing PSCP after prophylactic surgery is between 0.5–2% [18-20].

## Conclusion

This is the first described case of primary peritoneal carcinoma presenting as a secondary colonic malignancy many years after risk-reducing surgery. This diagnosis was reached following investigation and surgical resection for symptoms related to the lower gastrointestinal tract. Despite risk-reducing surgery in the form of prophylactic mastectomy and bilateral oopherectomy, albeit in the context of breast cancer, a primary peritoneal serous carcinoma developed many years later. The authors emphasise that although risk-reducing surgery has been shown to confer a clear reduction in risk of developing breast, ovarian and peritoneal carcinoma, the possibility of a late presentation of peritoneal carcinoma remains.

## Competing interests

The author(s) declare that they have no competing interests.

## Authors' contributions

**MC **– literature review; writing of manuscript and revisions; covering letters and addressing reviewers' comments. **PJM **– case-note review. **GFN **– conceived study, manuscript revision with particular reference to language and style. **ADC **– conceived study and helped review manuscript, **TH **– review of manuscript.

All authors read and approved the manuscript.

## References

[B1] Struewing JP, Hartge P, Wacholder S, Baker SM, Berlin M, McAdams M, Timmerman MM, Brody LC, Tucker MA (1997). The risk of cancer associated with specific mutations of *BRCA1 *and *BRCA2 *among Ashkenazi Jews. N Engl J Med.

[B2] Ford D, Easton DF, Stratton M, Narod S, Goldgar D, Devilee P, Bishop DT, Weber B, Lenoir G, Chang-Claude J, Sobol H, Teare MD, Struewing J, Arason A, Scherneck S, Peto J, Rebbeck TR, Tonin P, Neuhausen S, Barkardottir R, Eyfjord J, Lynch H, Ponder BA, Gayther SA, Zelada-Hedman (1998). Genetic heterogeneity and penetrance analysis of the *BRCA1 *and *BRCA2 *genes in breast cancer families. Am J Hum Genet.

[B3] Satagopan JM, Offit K, Foulkes W, Robson ME, Wacholder S, Eng CM, Karp SE, Begg CB (2001). The lifetime risks of breast cancer in Ashkenazi Jewish carriers of *BRCA1 *and *BRCA2 *mutations. Cancer Epidemiol Biomarkers Prev.

[B4] Altaras MM, Aviram R, Cohen I, Cordoba M, Weiss E, Beyth Y (1991). Primary peritoneal papillary serous adenocarcinoma: clinical and management aspects. Gynaecol Oncol.

[B5] Piura B, Rabinovich A, Yanai-Inbar I (2001). Three primary malignancies related to BRCA mutation successively occurring in a *BRCA1 *185delAG mutation carrier. Eur J Obstet Gynaecol Reprod Biol.

[B6] Levine DA, Argenta PA, Yee CJ, Marshall DS, Olvera N, Bogomolniy F, Rahaman JA, Robson ME, Offit K, Barakat RR, Soslow RA, Boyd J (2003). Fallopian tube and primary peritoneal carcinomas associated with *BRCA *mutations. J Clin Oncol.

[B7] Anonymous (2000). Prevalence and penetrance of *BRCA1 *and *BRCA2 *mutations in a population-based series of breast cancer cases. Anglian Breast Cancer Study Group. Br J Cancer.

[B8] Whittemore AS, Gong G, John EM, McGuire V, Li FP, Ostrow KL, Dicioccio R, Felberg A, West DW (2004). Prevalence of *BRCA1 *mutations carriers among non-Hispanic Whites. Cancer Epidemiol Biomarkers Prev.

[B9] Liede A, Karlan BY, Baldwin RL, Platt LD, Kuperstein G, Narod SA (2002). Cancer incidence in a population of Jewish women at risk of ovarian cancer. J Clin Oncol.

[B10] Finch A, Beiner M, Lubinski J, Lynch HT, Moller P, Rosen B, Murphy J, Ghadirian P, Freidman E, Foulkes WD, Kim-Sing C, Wagner T, Tung N, Couch F, Stoppa-Lyoneet D, Ainsworth P, Daly M, Pasini B, Gershoni-Baruch R, Eng C, Olopade OI, McLennan J, Karlan B, Weitzel J, Sun P, Narod SA (2006). Salpingo-oophorectomy and the risk of ovarian, Fallopian tube, and peritoneal cancers in women with a *BRCA1 *or *BRCA2 *mutation. JAMA.

[B11] Tobacman JK, Greene MH, Tucker MA, Costa J, Kase R, Fraumeni JF (1982). Intra-abdominal carcinomatosis after prophylactic oophorectomy in ovarian cancer-prone families. Lancet.

[B12] Kannerstein M, Churg J, McCaughey WT, Hill DP (1977). Papillary tumour of the peritoneum in women: mesothelioma or papillary carcinoma. Am J Obstet Gynecol.

[B13] Lauchlan SC (1972). The second mullerian system. Obstet Gynecol Surv.

[B14] Stafford-Johnson DB, Bree RL, Francis IR, Korobkin M (1998). CT appearance of primary papillary serous carcinoma of the peritoneum. AJR Am J Roentgenol.

[B15] Thompson D, Easton DF, Breast Cancer Linkage Consortium (2002). Cancer incidence in *BRCA1 *mutation carriers. J Natl Cancer Inst.

[B16] Breast Cancer Linkage Consortium (1999). Cancer risks in *BRCA2 *mutation carriers. J Natl Cancer Inst.

[B17] Casey MJ, Bewtra C (2004). Peritoneal carcinoma in women with genetic susceptibility: implications for Jewish populations. Fam Cancer.

[B18] Kauff ND, Satagopan JM, Robson ME, Scheuer L, Hensley M, Hudis CA, Ellis NA, Boyd J, Borgen PI, Barakat RR, Norton L, Castiel M, Nafa K, Offit K (2002). Risk-reducing salpingo-oophorectomy in women with a *BRCA1 *or *BRCA2 *mutation. N Engl J Med.

[B19] Piver MS, Jishi MF, Tsukada Y, Nava G (1993). Primary peritoneal carcinoma after prophylactic oophorectomy in women with a family history of ovarian cancer: A report of the Gilda Radner Familial Ovarian Cancer Registry. Cancer.

[B20] Rebbeck TR, Lynch HT, Neuhausen SL, Narod SA, Van't Veer L, Garber JE, Evans G, Isaacs C, Daly MB, Matloff E, Olopade OI, Weber BL (2002). Prevention and Observation of Surgical End Points Study Group. Prophylactic oophorectomy in carriers of *BRCA1 *and *BRCA2 *mutations. N Engl J Med.

